# Supplementary material for: Genomic signatures of pre-resistance in Mycobacterium tuberculosis
Source: Nat Commun. 2021 Dec 15;12:7312. doi: 10.1038/s41467-021-27616-7 (PMC8674244; doi:10.1038/s41467-021-27616-7)
Supplement: Supplementary file 1 — Supplementary Information [file 41467_2021_27616_MOESM1_ESM.pdf]

Supplementary information

Supplementary Table 1: Drug resistance profiles by project.

Drug profile[1,2]	Dataset				
	**Population**	**Household**	**CRyPTIC**	**Tandem**	**Other**
**Susceptible**	73.4% (1496/2037)	3.9% (7/180)	15.9% (77/483)	81.5% (327/401)	36.4% (12/33)
**DR**	14.1% (288/2037)	5.6% (10/180)	20.1% (97/483)	10% (40/401)	9.1% (3/33)
**Rifampicin**	1.6% (32/2037)	2.2% (4/180)	3.1% (15/483)	0.7% (3/401)	0% (0/33)
**Isoniazid**	5.2% (105/2037)	1.1% (2/180)	7.9% (38/483)	2.5% (10/401)	3% (1/33)
**Ethambutol**	0.5% (10/2037)	0% (0/180)	0% (0/483)	0.2% (1/401)	0% (0/33)
**Streptomycin**	1.3% (26/2037)	0% (0/180)	0.8% (4/483)	1.5% (6/401)	0% (0/33)
**Other**	5.6% (115/2037)	2.2% (4/180)	8.1% (40/483)	4.2% (20/401)	6.1% (2/33)
**MDR**	12.3% (251/2037)	88.3% (159/180)	59.2% (286/483)	8.5% (34/401)	51.5% (17/33)
**XDR**	0.1% (2/2037)	2.2% (4/180)	4.8% (23/483)	0% (0/401)	3% (1/33)

[1] Estimation of drug resistance profile using drug resistance associated SNPs [33]
[2] DR, drug resistance to a single antibiotic or a combination of antibiotics not included in MDR (Other); MDR, multi-drug resistance, defined as co-occurrence of isoniazid and rifampicing resistance; XDR, extensively drug resistance, defined as MDR in addition to resistance to any fluoroquinolone (ciprofloxacin, moxifloxacin, or ofloxacin) and at least one of three injectable second-line drugs (amikacin, kanamycin, or capreomycin) [2]

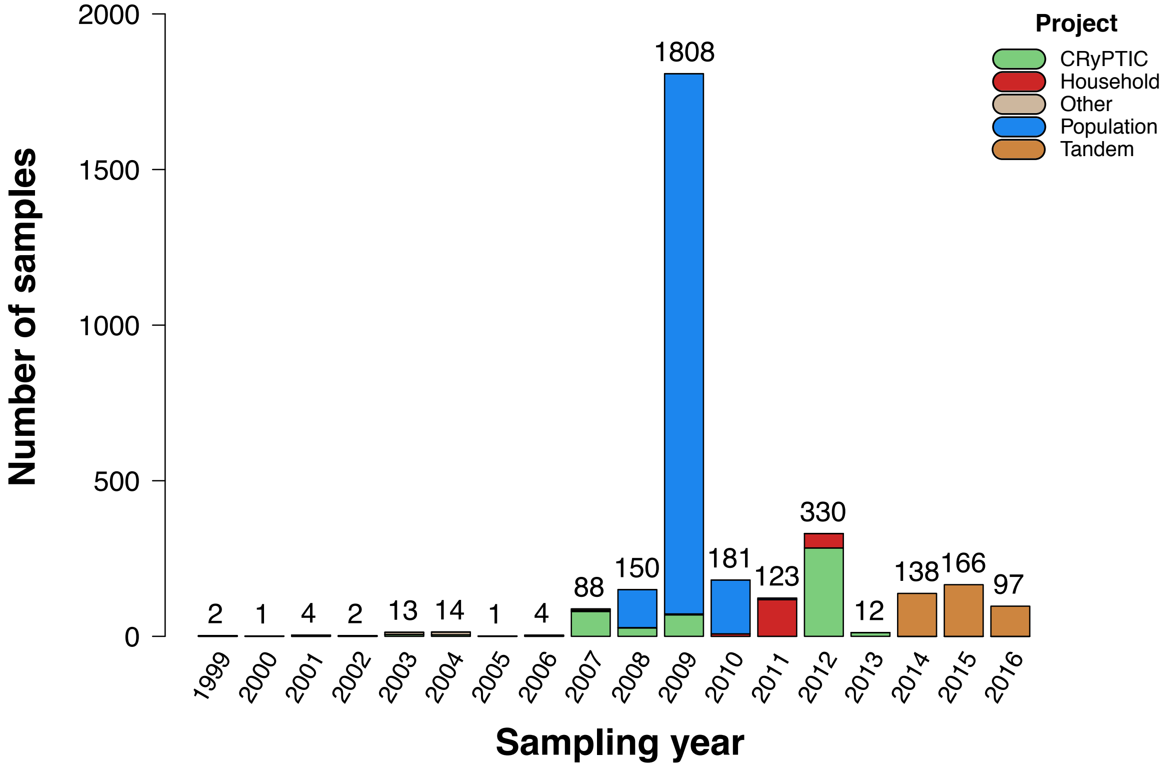

Supplementary Figure 1: Sample cohort
Temporal distribution of the 3134 samples included in the study.

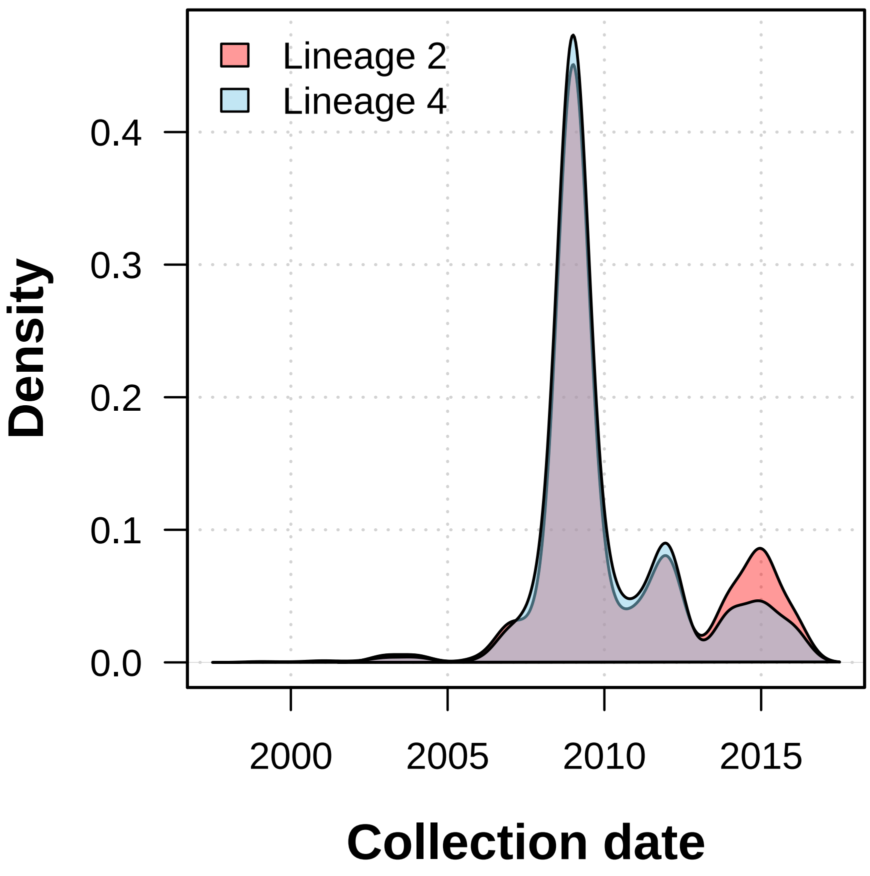

Supplementary Figure 2: Sampling date distribution
Distribution of sampling dates by lineage.

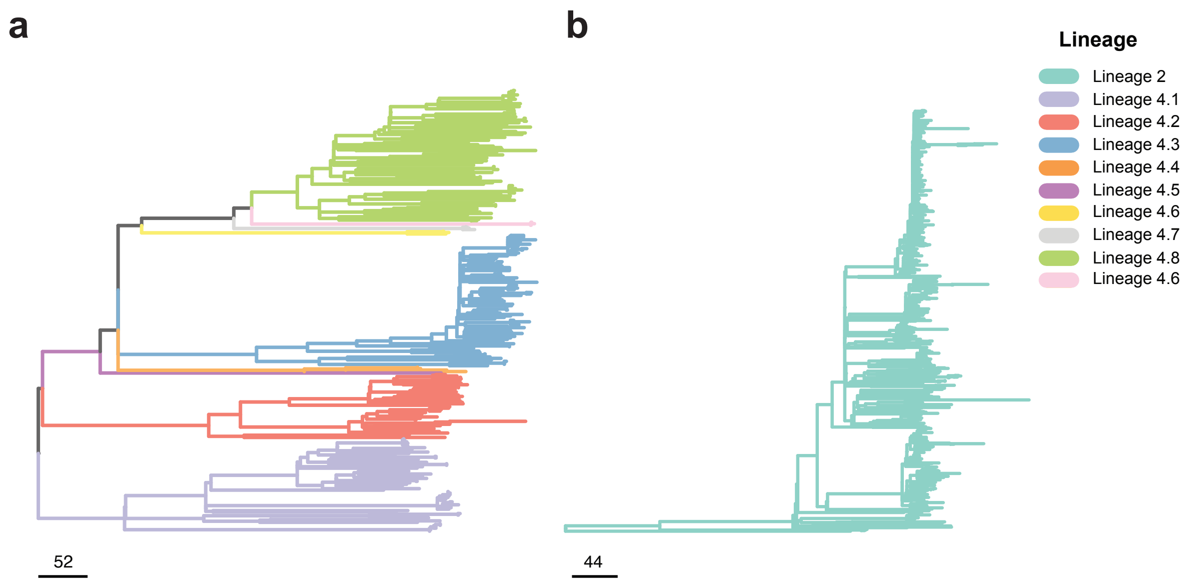

Supplementary Figure 3: Maximum likelihood phylogeny of the Samara (Russia) data set
Phylogeny of the Samara data set for **(a)** lineage 4 and **(b)** lineage 2.

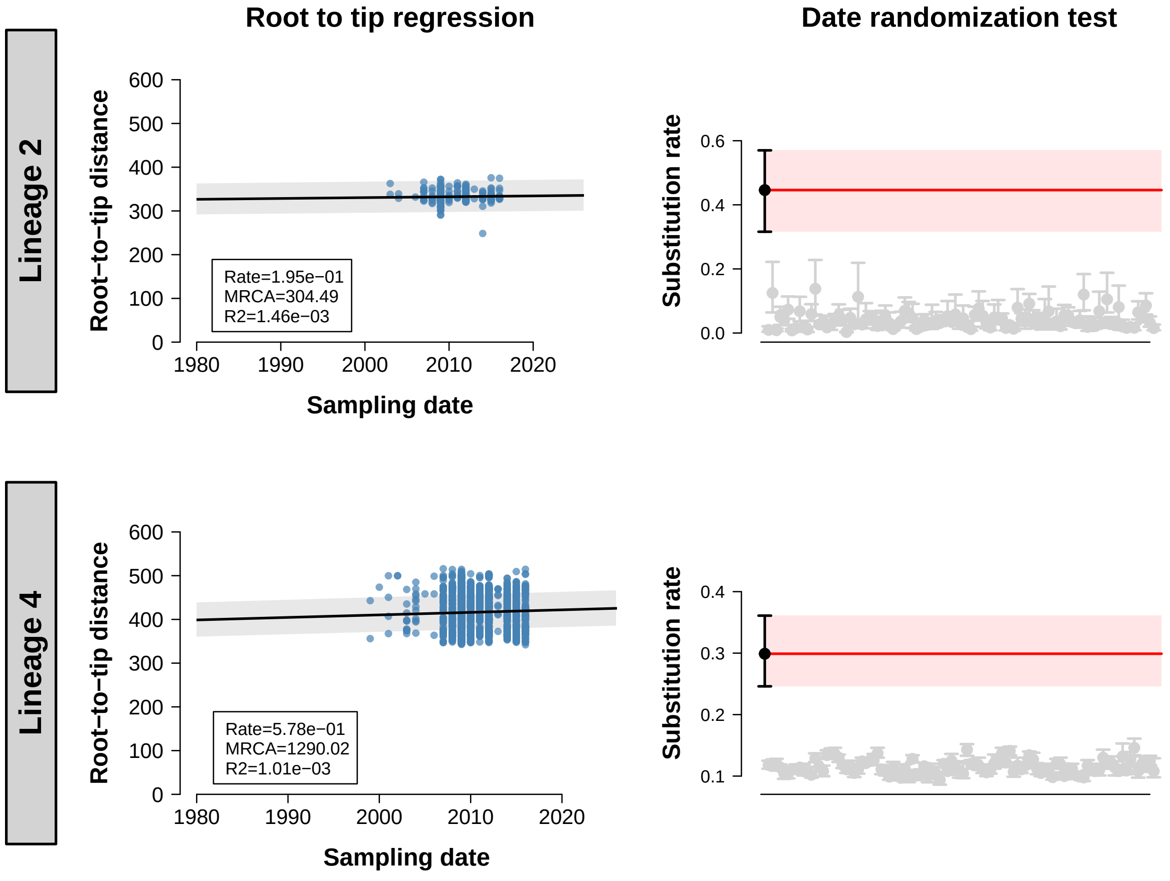

Supplementary Figure 4: Temporal signal analysis
The presence of measurable evolution (temporal signal) within the lineage 2 (top) and lineage 4 (bottom) was tested by a root-to-tip regression (left) and by a date-randomization test (right). In the root-to-tip regression the distance from the tips to the root of the phylogenetic tree (y-axis) are plotted against the sampling dates (x-axis). The mutation rate and the time of the most recent common ancestor (MRCA) are calculated from the linear regression. The solid black line represents the best fitting regression line while the grey shaded area shows the 95% CI estimated from a Gamma distribution. The date randomization test is performed by comparing the substitution rate of the original dataset (black) with a set of randomized data sets obtained by permutating the sampling dates (grey). The points show the mean of the distribution while error bars represent the 95% CI. The mean and 95% CI of the original dataset are also highlighted by a solid red line and a red shaded area, respectively. The date randomization test is passed if the estimates from the original data do not overlap with the randomized sets.

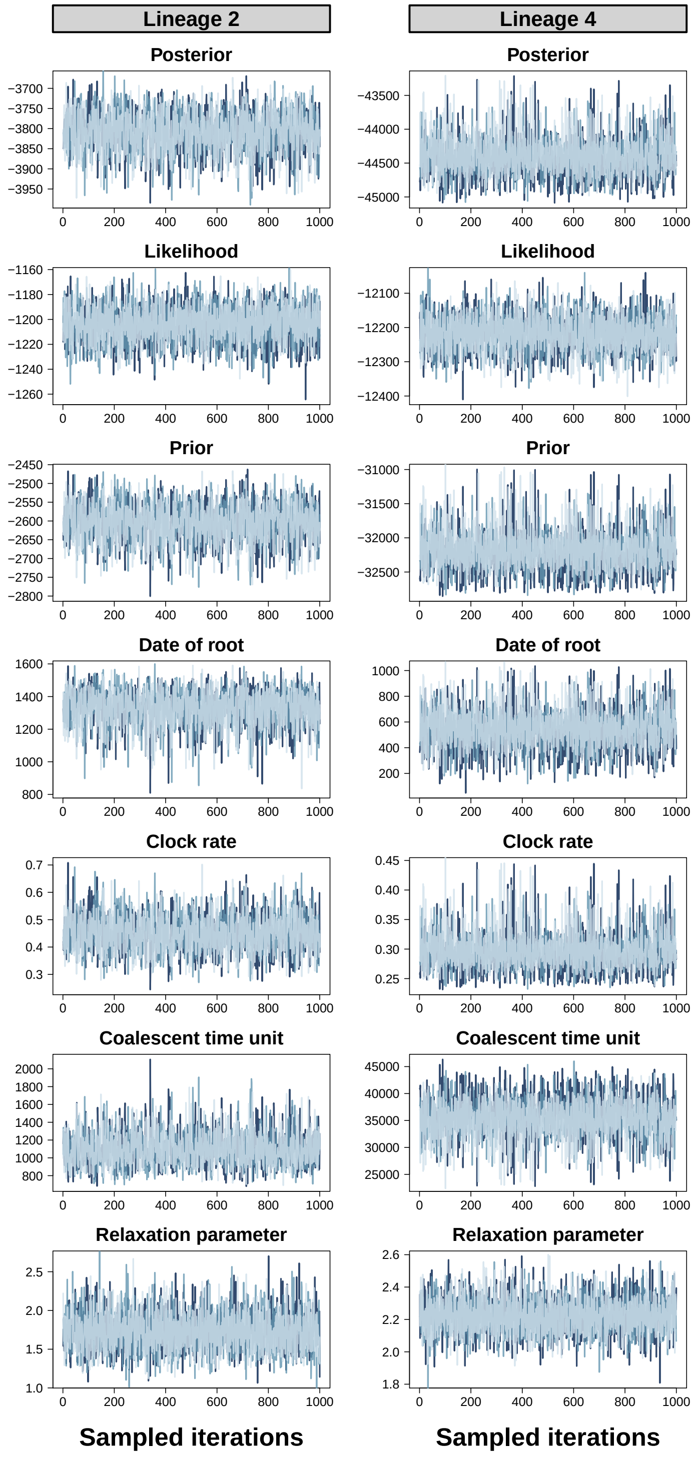

Supplementary Figure 5: BactDating MCMC chain convergence
BactDating trace output for lineage 2 and lineage 4 in three independent MCMC chains. Parameters shown are posterior probabilities, likelihood, prior probabilities, date of MRCA, substitution rates, coalescent time unit and relaxation parameters. Three independent chains were run for each dataset.

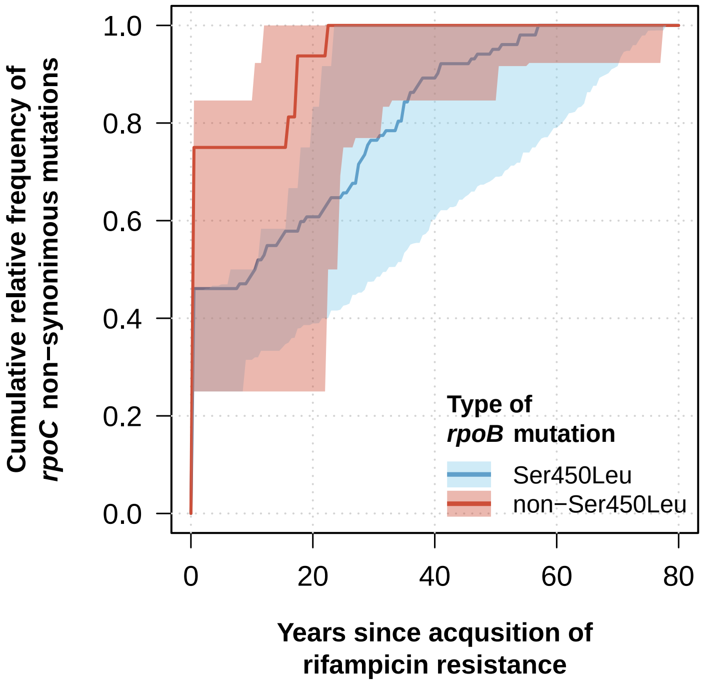

Supplementary Figure 6: Dynamics of non-synonymous mutations in the *rpoC* gene for Ser450Leu and non-Ser450Leu rpoB mutations
Cumulative relative frequency of non-synonymous mutations in *rpoC* over time. The x-axis represents the years since the inferred time of rifampcin resistance (time 0). Colors show mutations for Ser450Leu (blue) and non-Ser450Leu (red) *rpoB* rifampicin resistant isolates. Dark colors represent the number of mutations for the ML tree, while the confidence interval (shaded area) is inferred by repeating the analysis in 100 bootstrap phylogenies.

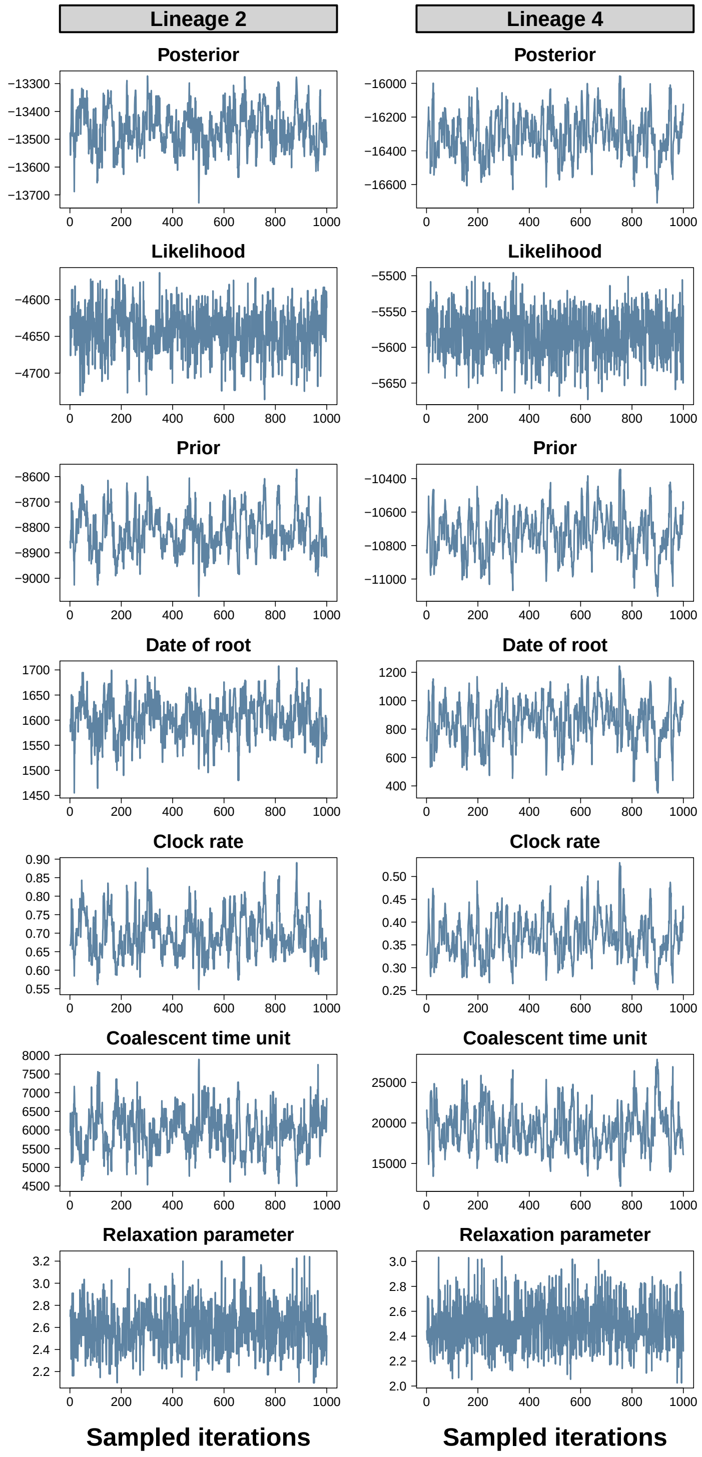

Supplementary Figure 7: BactDating MCMC chain convergence for the global data set

BactDating trace output for lineage 2 and lineage 4 in a global set of publicly available strains. Parameters shown are posterior probabilities, likelihood, prior probabilities, date of MRCA, substitution rates, coalescent time unit and relaxation parameters.

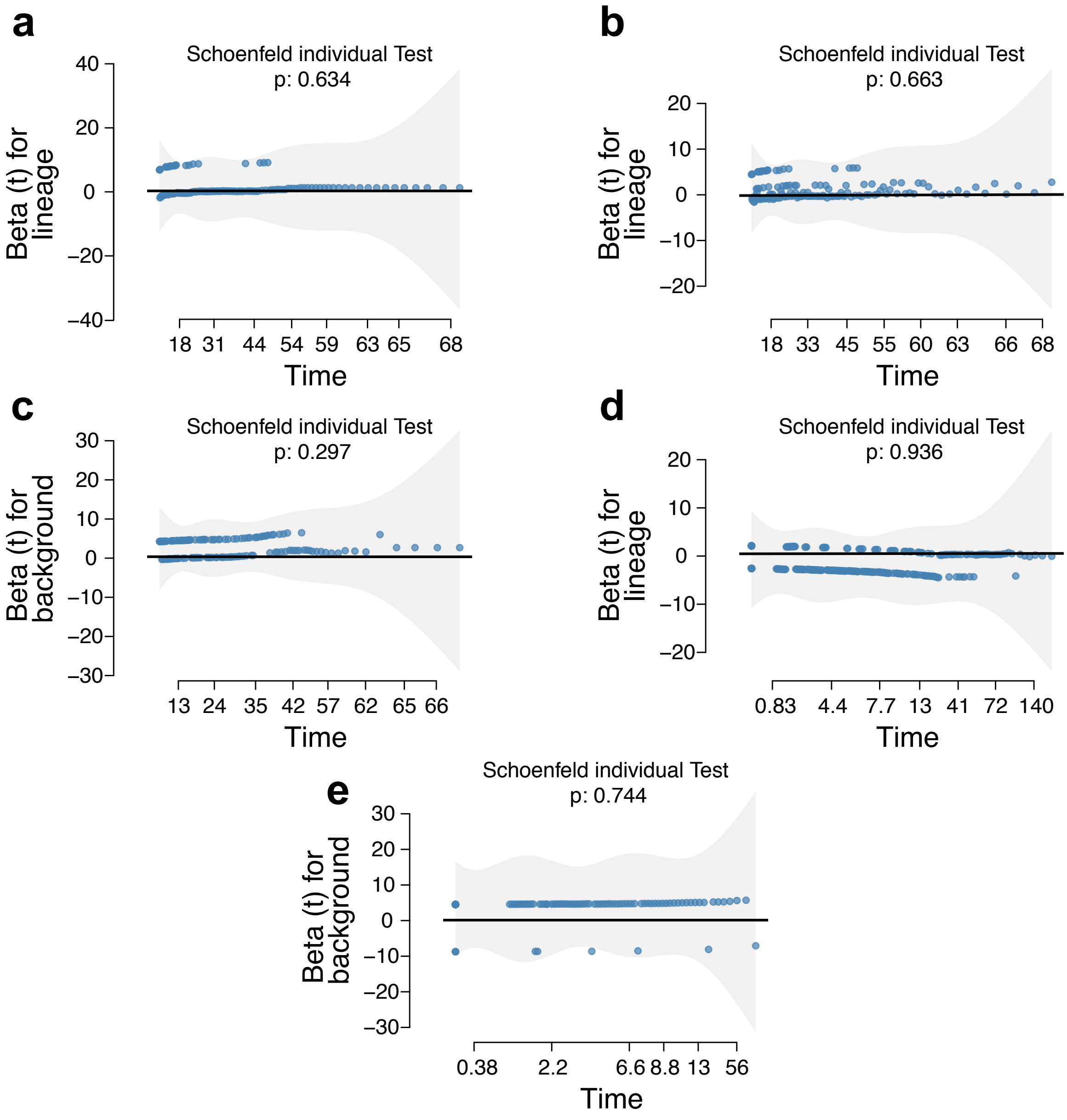

Supplementary Figure 8: Scaled Schoenfeld residuals
(a-e) Schoenfeld residuals plotted against time. In all cases, the proportional hazard assumption is supported by a non-significant association between residuals and time, thus the proportional hazards model can be assumed. The black solid line represents the best fitting line, while the grey shaded area shows the 95% CI for the regression. Test and plots modified from the *survminer* R package. **(a)** Residuals for lineage 2 and lineage 4 in Peru. **(b)** Residuals for lineage 2 and several sublineages of lineage 4 in Peru. **(c)** Residuals for sensitive and isoniazid mono-resistant background in Peru. **(d)** Residuals for lineage 2 and lineage 4 in Samara. **(e)** Residuals for sensitive and isoniazid mono-resistant background in Samara.

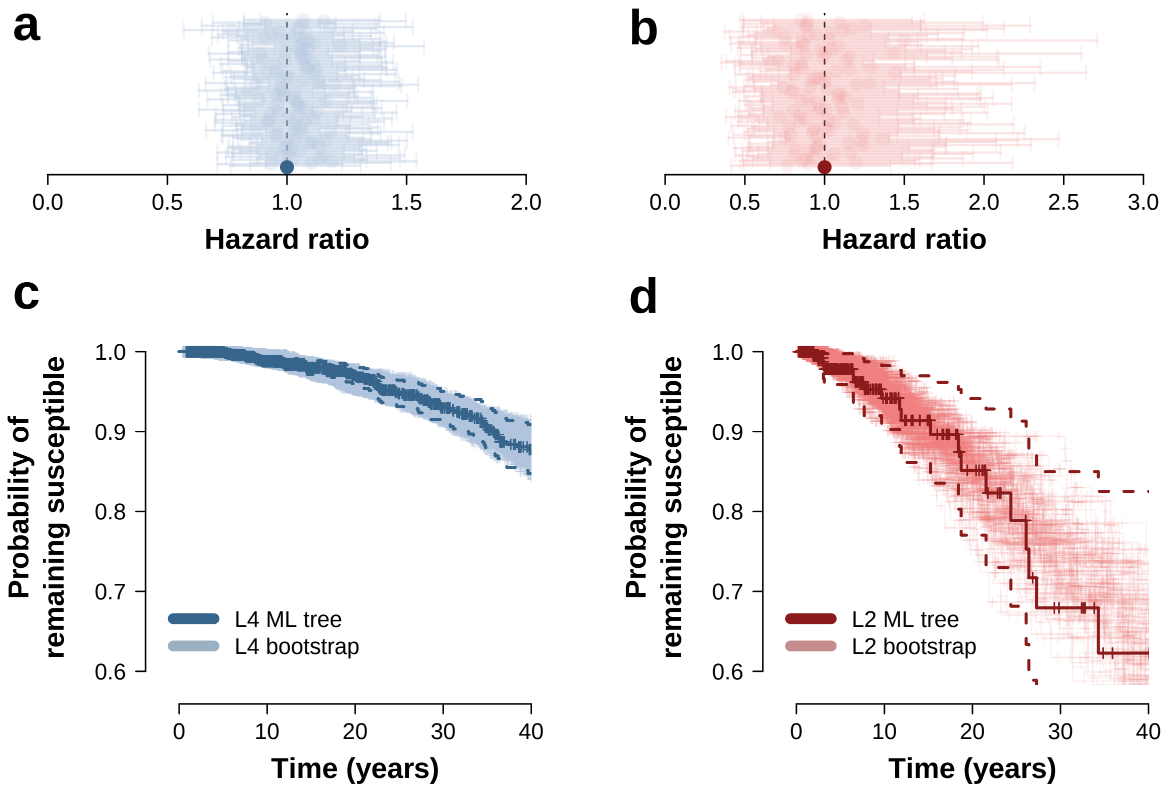

Supplementary Figure 9: Hazard ratio and Kaplan-Meier curve of 100 phylogenetic bootstrap replicates
100 phylogenetic bootstrap replicates were performed in parallel to the maximum likelihood phylogeny. Blue color represents the lineage 4 phylogeny while the red color shows the lineage 2 phylogeny. No statistical differences are found between the results obtained using the maximum likelihood tree and those using the phylogenetic bootstrap replicates. **(a, b)**, Hazard ratio for the maximum likelihood tree (dark point) and 100 phylogenetic bootstrap replicates (light colored points). Points and error bars represent the HR estimate and the 95% CI, respectively. **(c, d)**, Kaplan-Meier curve for the maximum likelihood tree (dark solid lines) with the 95% CI interval (dark dotted lines), and 100 phylogenetic bootstrap replicates (light colored lines).

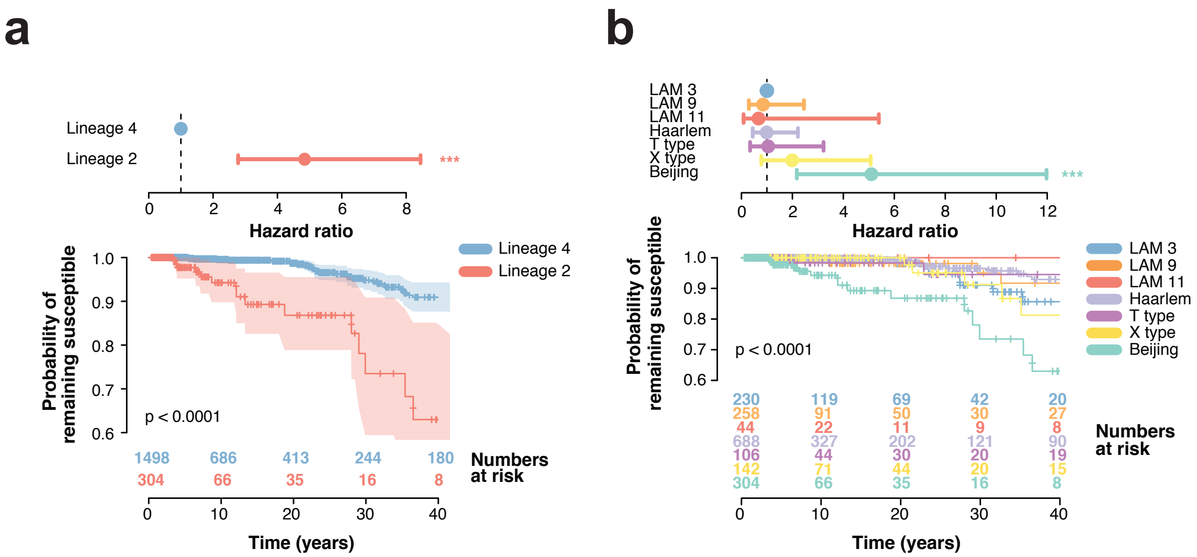

Supplementary Figure 10: Hazard ratio and Kaplan-Meier curve for drug resistance acquisition in the population level dataset
(a-b) Top: Hazard ratio (HR). Points and error bars represent the HR estimate and the 95% CI, respectively. The p-value for the HR was calculated using the likelihood ratio test. Bottom: Kaplan-Meier curve and numbers at risk. Y-axis represents the probability of remaining susceptible to any antibiotic, while the X-axis shows the time in years or the distance in branch length. Shaded areas show the 95% confidence interval. P-values for the Kaplan-Meier curves were calculated using the log-rank test. **(a)** HR of lineage 2 compared to lineage 4 in the Peruvian population level dataset (HR 4.84, 95% CI 2.78-8.45, Likelihood ratio test p-value $= 2.7 \times 10^{-8}$) and the different Kaplan-Meier curve for lineage 2 and lineage 4 (log-rank test p-value $= 7.9 \times 10^{-10}$). **(b)** HR between lineage 2 and the different sublineages of lineage 4 found in the Peruvian dataset (LAM9, LAM3, LAM11, Haarlem, X type and T type), using LAM3 as a reference (lineage 2 HR 5.1, 95% CI 2.17-11.9, Likelihood ratio test p-value $= 1.8 \times 10^{-4}$, all other p-values > 0.2; Kaplan-Meier curve Log-rank test p-value $= 3.02 \times 10^{-7}$). Statistical significance of the hazard ratio differences presented next to the CI bars (*, p<0.05; **, p<0.01; ***, p<0.001)

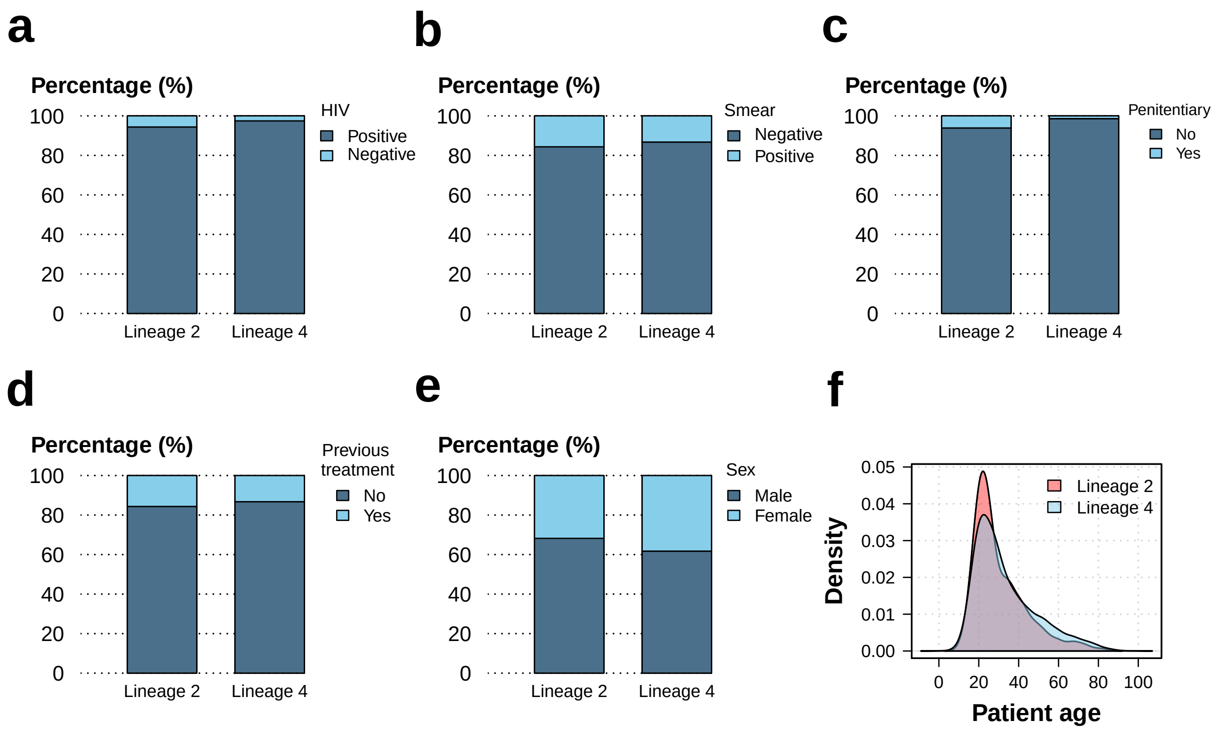

Supplementary Figure 11: ***Mycobacterium tuberculosis*** **lineage differences in non-bacterial factors.**
Distribution of environmental factors in lineage 2 and lineage 4. **(a)**, HIV status. **(b)**, Smear positivity. **(c)**, Imprisonment status. **(d)**, Previous treatment with anti-tubercular drugs. **(e)**, Patient sex. **(f)**, Distribution of patients age.

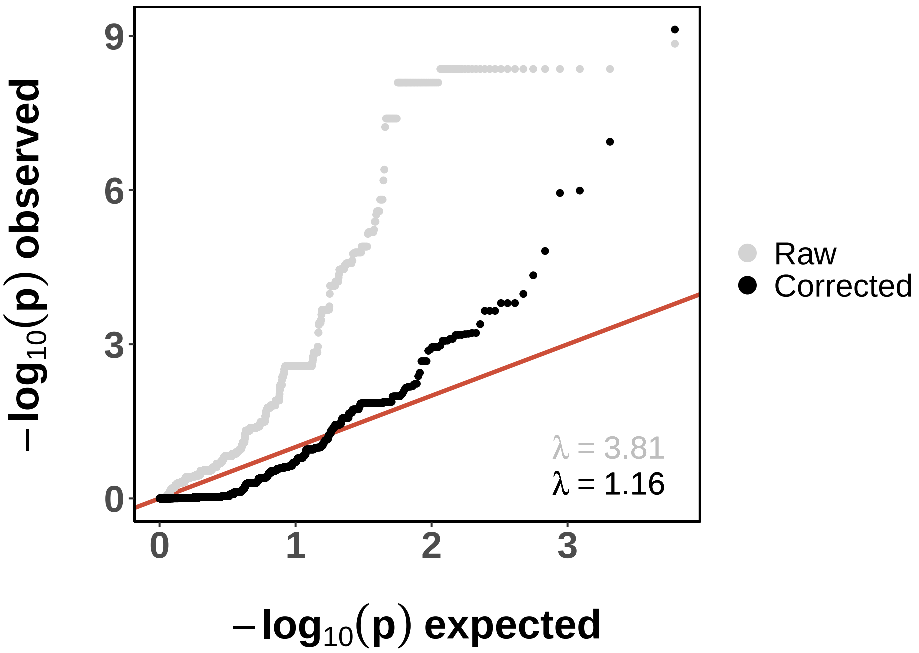

Supplementary Figure 12: Population correction for Genome-Wide association study (GWAS)
Quantile-quantile (QQ) for the raw p-values (grey), and the p-values corrected for population structure (black). Red line indicates the null hypothesis of uniformly distributed p-values. λ represents the genomic inflation factor.

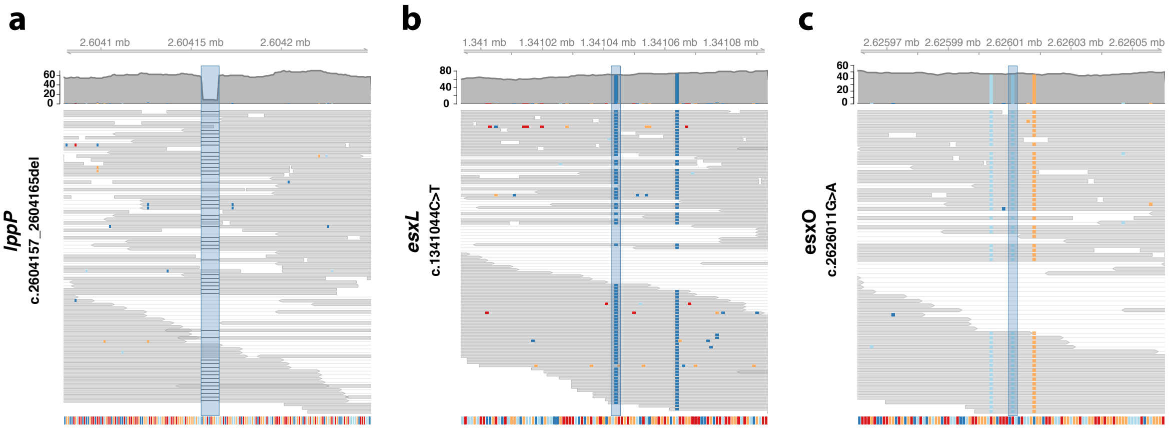

Supplementary Figure 13: Short-read alignment around GWAS polymorphisms

Coverage plot (top) and alignments from paired-end reads around GWAS hits. Alignments (middle panel) are represented as gray polygons with mismatches from the reference genome (bottom) indicated by different colors. Gaps are shown as black bars. Shaded area highlights the variant of interest. **(a)** *lppP* deletion. **(b)** SNP in the *esxL* gene, showing a T instead of the reference C. **(c)** Base mutation from G to A in the *esxO* gene.
