# Supplementary material for: Genomic signatures of pre-resistance in Mycobacterium tuberculosis
Source: Nat Commun. 2021 Dec 15;12:7312. doi: 10.1038/s41467-021-27616-7 (PMC8674244; doi:10.1038/s41467-021-27616-7)
Supplement: Supplementary file 3 — Description of Additional Supplementary Files [file 41467_2021_27616_MOESM3_ESM.pdf]

Description of Additional Supplementary Files

File Name: Supplementary Data 1
Description: Accession code and collection date of *M. tuberculosis* whole-genome sequences collected at the population level in Lima, Peru.

File Name: Supplementary Data 2
Description: Accession codes, collection date and geographical metadata of *M. tuberculosis* global representatives.

File Name: Supplementary Data 3
Description: Accession codes and collection date of *M. tuberculosis* lineage 4 global data set.
