# Supplementary material for: Genomic signatures of pre-resistance in Mycobacterium tuberculosis
Source: Nat Commun. 2021 Dec 15;12:7312. doi: 10.1038/s41467-021-27616-7 (PMC8674244; doi:10.1038/s41467-021-27616-7)
Supplement: Supplementary file 7 — Reporting Summary [file 41467_2021_27616_MOESM7_ESM.pdf]

nature research

Corresponding author(s): Dr. Louis Grandjean

Last updated by author(s): Nov 10, 2021

Reporting Summary

Nature Research wishes to improve the reproducibility of the work that we publish. This form provides structure for consistency and transparency in reporting. For further information on Nature Research policies, see our Editorial Policies and the Editorial Policy Checklist.

Statistics

For all statistical analyses, confirm that the following items are present in the figure legend, table legend, main text, or Methods section.

n/a	Confirmed	
☐	☒	The exact sample size (*n*) for each experimental group/condition, given as a discrete number and unit of measurement
☐	☒	A statement on whether measurements were taken from distinct samples or whether the same sample was measured repeatedly
☐	☒	The statistical test(s) used AND whether they are one- or two-sided<br>*Only common tests should be described solely by name; describe more complex techniques in the Methods section.*
☐	☒	A description of all covariates tested
☐	☒	A description of any assumptions or corrections, such as tests of normality and adjustment for multiple comparisons
☐	☒	A full description of the statistical parameters including central tendency (e.g. means) or other basic estimates (e.g. regression coefficient) AND variation (e.g. standard deviation) or associated estimates of uncertainty (e.g. confidence intervals)
☐	☒	For null hypothesis testing, the test statistic (e.g. *F*, *t*, *r*) with confidence intervals, effect sizes, degrees of freedom and *P* value noted<br>*Give P values as exact values whenever suitable.*
☐	☒	For Bayesian analysis, information on the choice of priors and Markov chain Monte Carlo settings
☐	☒	For hierarchical and complex designs, identification of the appropriate level for tests and full reporting of outcomes
☐	☒	Estimates of effect sizes (e.g. Cohen's *d*, Pearson's *r*), indicating how they were calculated

Our web collection on statistics for biologists contains articles on many of the points above.

Software and code

Policy information about availability of computer code

Data collection	ENA links were generated using the accession codes and downloaded with the wget within BASH.
Data analysis	FastQC, SPAdes v3.14.0, minimap2, BCFtools v1.9, BWA v0.7.17, SAMtools v1.9, Picard v2.19.0, GATK v3.8-1-0, Python v3.7.3, VEP v104.3, RAxML-NG, R, BactDating 1.1.0, Phangorn 2.7.1, Ape 5.5, Surviva 3.2-13l, phytools 0.7-90. Custom code: https://github.com/arturotorreso/mtb_pre-resistance

For manuscripts utilizing custom algorithms or software that are central to the research but not yet described in published literature, software must be made available to editors and reviewers. We strongly encourage code deposition in a community repository (e.g. GitHub). See the Nature Research guidelines for submitting code & software for further information.

Data

Policy information about availability of data

All manuscripts must include a data availability statement. This statement should provide the following information, where applicable:

- Accession codes, unique identifiers, or web links for publicly available datasets
- A list of figures that have associated raw data
- A description of any restrictions on data availability

All raw sequencing data are available with accession numbers listed in Material and Methods. Samples sequenced as part of this study have been submitted to the European Nucleotide Archive under accession PRJEB39837 and PRJEB47846. Publicly available datasets used in this study include PRJEB5280, PRJEB32234, PRJEB23245, and PRJEB2138. All other publicly available datasets are listed in Supplementary Data File 2 and Supplementary Data File 3.

Field-specific reporting

Please select the one below that is the best fit for your research. If you are not sure, read the appropriate sections before making your selection.

☒ Life sciences ☐ Behavioural & social sciences ☐ Ecological, evolutionary & environmental sciences

For a reference copy of the document with all sections, see nature.com/documents/nr-reporting-summary-flat.pdf

Life sciences study design

All studies must disclose on these points even when the disclosure is negative.

Sample size	All samples were collected as part of a population level study carried out in 2009 where sputum samples were taken from all patients presenting tuberculosis symptoms in the Lima areas of Callao and Lima South.
Data exclusions	Samples with low quality genome sequence data or absence of collection date were excluded from the analysis.
Replication	The analysis was replicated in a publicly available data set.
Randomization	Samples were collected from all patients presenting tuberculosis symptoms at the health centers.
Blinding	No group assignments were required for this project.

Reporting for specific materials, systems and methods

We require information from authors about some types of materials, experimental systems and methods used in many studies. Here, indicate whether each material, system or method listed is relevant to your study. If you are not sure if a list item applies to your research, read the appropriate section before selecting a response.

Materials & experimental systems

n/a	Involved in the study	
☒	☐	Antibodies
☒	☐	Eukaryotic cell lines
☒	☐	Palaeontology and archaeology
☒	☐	Animals and other organisms
☒	☐	Human research participants
☒	☐	Clinical data
☒	☐	Dual use research of concern

Methods

n/a	Involved in the study	
☒	☐	ChIP-seq
☒	☐	Flow cytometry
☒	☐	MRI-based neuroimaging
